# Maternal Autistic Traits and Adverse Birth Outcomes

**DOI:** 10.1001/jamanetworkopen.2023.52809

**Published:** 2024-01-23

**Authors:** Mariko Hosozawa, Noriko Cable, Satoyo Ikehara, Yuri Aochi, Kanami Tanigawa, Sachiko Baba, Kumi Hirokawa, Tadashi Kimura, Tomotaka Sobue, Hiroyasu Iso

**Affiliations:** 1 Institute for Global Health Policy Research, Bureau of International Health Cooperation, National Center for Global Health and Medicine, Tokyo, Japan; 2 Department of Epidemiology and Public Health, University College London, London, United Kingdom; 3 Environmental Medicine and Population Sciences, Department of Social Medicine, Osaka University Graduate School of Medicine, Osaka, Japan; 4 Osaka Maternal and Child Health Information Center, Osaka Women’s and Children’s Hospital, Osaka, Japan; 5 Faculty of Societal Safety Sciences, Kansai University, Osaka, Japan; 6 Department of Obstetrics and Gynecology, Osaka University Graduate School of Medicine, Osaka, Japan; 7 Public Health, Department of Social Medicine, Osaka University Graduate School of Medicine, Osaka, Japan

## Abstract

**Importance:**

Women with a high level of autistic traits in the general population may experience larger health disparities during pregnancy, particularly women diagnosed with autism spectrum disorder (ASD), which in turn may be associated with increased risk of adverse birth outcomes.

**Objective:**

To investigate the association between maternal autistic traits and the risk of adverse birth outcomes in the general population.

**Design, Setting, and Participants:**

This cohort study included mothers of singletons from a nationwide, multicenter prospective birth cohort, the Japan Environmental Children’s Study. Expecting mothers were recruited between January 2011 and March 2014. Data were analyzed between June 2021 and November 2023.

**Exposures:**

Autistic traits were self-reported during the second and third trimesters using the short form of the Autism-Spectrum Quotient Japanese version (AQ-J10) (score range, 0-10; clinical range, ≥7).

**Main Outcomes and Measures:**

Data on preterm birth (<37 weeks’ gestation) and neonates born small for gestational age (SGA) were transcribed from medical records. Additional analysis of gestational age groups (very preterm birth, <32 weeks’ gestation; moderate-to-late preterm birth, 32-36 weeks’ gestation) was also performed.

**Results:**

Among 87 687 women (mean [SD] age, 31.2 [5.0] years) included in the study, 2350 (2.7%) had AQ-J10 scores within the clinical range yet only 18 (0.02%) were diagnosed with ASD. A higher AQ-J10 score was associated with an increased risk of all birth outcomes, including preterm births (relative risk [RR] per 1-SD increase, 1.06; 95% CI, 1.03-1.09), moderate-to-late preterm births (RR per 1-SD increase, 1.05; 95% CI, 1.01-1.08), very preterm births (RR per 1-SD increase, 1.16; 95% CI, 1.06-1.26), and child born SGA (RR per 1-SD increase, 1.04; 95% CI, 1.01-1.06) after adjusting for maternal and pregnancy-related factors. The risks of all outcomes increased with higher AQ-J10 scores; compared with women below the clinical range, women within the clinical range had greater risk of preterm births (RR, 1.16; 95% CI, 1.07-1.26), moderate-to-late preterm births (RR, 1.12; 95% CI, 1.03-1.22), very preterm births (RR, 1.49; 95% CI, 1.18-1.89), and a child born SGA (RR, 1.11; 95% CI, 1.04-1.19).

**Conclusions and Relevance:**

In this cohort study, higher level of maternal autistic traits was associated with increased risk of adverse birth outcomes, particularly very preterm birth. Acknowledging the risks and providing tailored and timely antenatal care support to women with a high level of autistic traits in the general population, particularly women with autistic traits within the clinical range, regardless of formal diagnosis, is warranted.

## Key Points

**Question:**

Are self-reported maternal autistic traits measured during the second or third trimester in the general population associated with adverse birth outcomes?

**Findings:**

In this cohort study of 87 687 women, a higher level of maternal autistic traits was associated with increased risk of preterm birth, particularly very preterm birth and small for gestational age. These risks were most pronounced for women within the clinical range, although most of them lacked a formal diagnosis of autism spectrum disorder.

**Meaning:**

Women with a high level of autistic traits in the general population, particularly those with autistic traits in the clinical range, may have an increased risk of adverse birth outcomes, highlighting the need for tailored and timely antenatal care support for these women.

## Teaser

This cohort study assesses the association of maternal autistic traits with preterm birth and neonates born small for gestational age.

## Introduction

Autism spectrum disorder (ASD) is a lifelong neurodevelopmental condition characterized by differences in social communication and restricted-repetitive behavior.^1^ Recently, several studies have indicated that women with ASD may experience significant health disparities during pregnancy, including inadequate access to health care,^2^ multiple chronic health conditions,^3,4^ higher levels of antenatal psychological distress,^5,6^ and greater risk of pregnancy complication,^7^ all of which are associated with increased risk of adverse birth outcomes, such as preterm birth and child born small for gestational age (SGA).^8,9,10^ Despite women with ASD being susceptible to experiencing adverse birth outcomes, only a few studies have examined birth outcomes in this population. One study based on Swedish medical records reported an increased risk of preterm birth among women diagnosed with ASD.^7^ Two other studies reported an increased risk of preterm birth among women with intellectual and developmental disabilities including ASD,^11,12^ but the associations specific to ASD were not available, as various conditions were treated together. Furthermore, previous studies have focused only on women diagnosed with ASD.^7,11,12^ However, ASD is increasingly viewed as a spectrum in which autistic traits are continuously distributed across the general population, and individuals diagnosed with ASD score at the end of the distribution.^13,14^ It has been reported that individuals who have a high level of subclinical autistic traits may share similar genetic features^14,15^ and social and psychological difficulties with those diagnosed with ASD.^16,17^ Given that women with an elevated level of autistic traits are less likely to receive a formal diagnosis than men,^18,19^ which may create further challenges in gaining support,^20^ there is a need to investigate the association between maternal autistic traits in the general population and adverse birth outcomes. This approach will help identify women who need additional support during pregnancy regardless of having received a formal diagnosis of ASD, with the potential to improve maternal and child outcomes.^21,22,23^

Using data from a nationwide cohort in Japan, this study aimed to examine the association between maternal autistic traits measured during the second and third trimesters and birth outcomes (preterm birth and child born SGA). Based on the results of previous studies,^7,11,12^ we hypothesized that maternal autistic traits measured using the short form of the Autism-Spectrum Quotient Japanese version (AQ-J10)^24^ would be associated with an increased risk of adverse birth outcomes and that this increase would be particularly pronounced for women with scores in the clinical range.

## Methods

### Study Population

This cohort study included participants from the Japan Environmental Children’s Study, a nationwide, multicenter, prospective birth cohort study funded by the Ministry of the Environment of Japan that recorded the health and development of children born by 103 060 pregnancies among women recruited between January 2011 and March 2014.^25^ Details of the study design are described elsewhere.^26,27^ Of the women with singleton live births, those with children with implausible birth weight for gestational age (±5 SDs) or gestational age older than 42 weeks and those with children with definitive chromosomal abnormalities reported by the physician at birth were excluded from the analysis. Cases with missing data on exposure, outcome, or covariates were also excluded (eFigure 1 in Supplement 1). This study was conducted according to guidelines in the Declaration of Helsinki.^28^ The Japan Environmental Children’s Study protocol was reviewed and approved by the Japan Ministry of the Environment’s Institutional Review Board on Epidemiological Studies, and the ethics committees of all participating institutions approved all procedures involving participants. Written informed consent was obtained from all the participants. This study followed the Strengthening the Reporting of Observational Studies in Epidemiology (STROBE) reporting guideline in reporting of the results.

### Maternal Autistic Traits

Maternal autistic traits were measured during the second or third trimesters using the AQ-J10.^24^ The AQ-J10 is a Japanese version of the internationally used self-reported questionnaire that quantifies autistic traits, mainly autistic social traits, in the general population.^24,29,30^ The questionnaire consists of 10 items adapted from the full 50-item AQ,^31,32^ which is a sensitive measure of autistic traits in the general population.^33,34^ Each item is answered on a 4-point Likert scale of “definitely agree,” “slightly agree,” “slightly disagree,” and “definitely disagree.” The responses were scored either 0 or 1 (responses “strongly agree” and “agree” would be given a score of 1 for responses that indicate autistic traits) to calculate the total score (range, 0-10). Higher total scores indicated a higher level of autistic traits. In the Japanese population, a score of 7 was recommended as the clinical threshold, indicating a potential need for full ASD assessment.^24^ The sensitivity and specificity of the AQ-J10 were reported as 0.76 and 0.90, respectively.^24^ The Cronbach α in our study was 0.52, indicating acceptable internal consistency.

### Birth Outcomes

Birth outcomes included preterm birth (<37 weeks’ gestation) and child born SGA (defined as birth weight less than the tenth percentile of the sex and birth-order specific means for gestational age in Japan).^35^ Preterm birth was further classified into moderate-to-late preterm birth (32-36 weeks’ gestation) and very preterm birth (<32 weeks’ gestation). All birth information was transcribed from the medical records at childbirth.

### Covariates

The following variables reported to be associated with the exposure and outcomes were included as covariates in the present study^3,4,8,9,10,36^: maternal age at childbirth, maternal highest level of education (categorized as a qualification of high school or below, vocational school or junior college, and university and higher), primiparous or not, smoking during pregnancy (yes vs no), prepregnancy maternal body mass index, preexisting physical health condition (eMethods in Supplement 1), and child sex (female or male). Furthermore, we included pregnancy complications (ie, absence or presence of gestational hypertension and gestational diabetes) for which the risk has been reported to be high among women with developmental and intellectual disabilities.^37^ Pregnancy complications, maternal age, and child sex were transcribed from medical records at childbirth, whereas all other information was obtained from maternal self-reports during pregnancy.

### Statistical Analyses

Statistical analyses were performed between June 2021 and November 2023. Descriptive analysis of the variables was performed. Sample bias analyses were used to compare whether the individuals excluded from the analysis differed from those included in this study. In the main analyses, we applied generalized linear models with Poisson distribution and a log-link function with robust SEs to obtain relative risks (RRs) for the association between maternal autistic traits and birth outcomes.^38^ Model 1 was a crude model, model 2 was adjusted for covariates (maternal age at childbirth, maternal educational level, primiparous or not, smoking during pregnancy, prepregnancy body mass index, preexisting physical health condition, and child sex), and model 3 was further adjusted for pregnancy complications (gestational hypertension and gestational diabetes). The effect estimates are presented per 1-SD increase in maternal autistic traits to aid interpretation. To illustrate the observed association found in model 3, we performed postestimation analysis and obtained the estimated marginal probability of the outcomes given all other variables at their mean by each level of maternal autistic traits using the Stata MP, version 18 (StataCorp LLC) command margins and graphically presented it using the command marginsplot. To quantify the risk of adverse birth outcomes for women scoring in the clinical range compared with those scoring below, we derived relative risks comparing women scoring above vs below the clinical threshold for the AQ-J10 using estimates from model 3. For this analysis, we first obtained mean risks of outcomes in each group using the postestimation Stata command margins and then estimated RRs using the Stata command nlcom, which uses the delta method for calculation.^39^ All analyses were repeated using gestational age subgroups (very preterm, <32 weeks’ gestation; moderate-to-late preterm, 32-36 weeks’ gestation) as outcomes to examine whether the association differed according to gestational age groups.

We conducted 2 sensitivity analyses. First, we adjusted for antenatal psychological distress measured using the Japanese version of the Kessler Psychological Distress Scale^40^ administered during the first trimester to confirm that the association between maternal autistic traits and the outcomes were not confounded by preceding antenatal psychological distress. Second, to confirm that the association observed was not influenced by preexisting psychiatric conditions or psychotropic medication receipt during pregnancy,^41,42^ we repeated the analyses excluding women reporting a history of psychiatric conditions or women who used psychotropic medication during pregnancy (eMethods in Supplement 1).

The proportion of missing data was considerably small in the present study (5.0%); therefore, the results of a complete case analysis are reported. All analyses were performed using Stata MP, version 18.0. Tests of statistical significance were based on 2-tailed *P* values or 95% CIs, and statistical significance was set at *P* < .05.

## Results

Of 92 944 women with singleton live births in the Japan Environmental Children’s Study, 13 were excluded because of their children having implausible birth weight for gestational age, 508 because gestational age was older than 42 weeks, and 144 because of chromosomal abnormalities. A total of 4592 women were excluded because of missing data on exposure, outcome, or covariates, leaving 87 687 women for the analysis. Participants’ descriptive characteristics are presented in Table 1. Of the 87 687 participating women (mean [SD] age, 31.2 [5.0] years), 38 370 (43.8%) were primiparous, 18 362 (20.9%) had preexisting physical health conditions, and 4645 (5.3%) had smoked during pregnancy. In the sample, the mean (SD) AQ-J10 score was 2.8 (1.7) and 2350 (2.7%) women scored above the clinical threshold; however, only 18 (0.02) were diagnosed with ASD. The distribution of maternal autistic traits is shown in eFigure 2 in Supplement 1. Women scoring within the AQ-J10 clinical range, compared with those scoring below the range, were more likely to be younger and primiparous and to have attained a lower educational level, smoked during pregnancy, and had higher antenatal psychological distress scores (eTable 1 in Supplement 1). The sample bias analyses comparing women included in the analysis with those excluded due to missing responses revealed that although there were no differences in mean AQ-J10 scores, women scoring in the clinical range were more likely to be excluded (eTable 2 in Supplement 1). Women who had lower educational levels, were primiparous, smoked during pregnancy, and had higher antenatal psychological distress scores were also more likely to be excluded from the study. In addition, more women with a preterm birth were excluded from this study; however, there were no significant group differences in the proportion with a child born SGA.

**Table 1.** Descriptive Characteristics of Study Participants

Characteristic	Participants (N = 87 687)^a^
Maternal age at delivery, mean (SD), y	31.2 (5.0)
Maternal autistic traits, mean (SD), No.^b^	2.8 (1.7)
Autistic traits in the clinical range^b^	2350 (2.7)
Highest maternal educational level	
High school graduate or below	31 529 (36.0)
Vocational school or junior college	36 957 (42.1)
College graduate and above	19 201 (21.9)
Primiparous	38 370 (43.8)
Prepregnancy body mass index, mean (SD)^c^	21.2 (3.3)
Preexisting physical health condition	18 362 (20.9)
Ever diagnosed with ASD	18 (0.02)
Smoked during pregnancy	4645 (5.3)
Gestational hypertension	2765 (3.2)
Gestational diabetes	2364 (2.7)
Child sex	
Female	42 710 (48.7)
Male	44 977 (51.3)
Antenatal psychological distress score, mean (SD)^d^	3.7 (3.8)

Abbreviation: ASD, autism spectrum disorder.

^a^ Data are presented as number (percentage) of participants unless otherwise indicated.

^b^ Measured using the short form of the Autism-Spectrum Quotient Japanese version administered during the second and third trimesters. The score ranges from 0 to 10, and the clinical range was a score at or above the clinical cutoff of 7.

^c^ Calculated as weight in kilograms divided by height in meters squared.

^d^ Measured using the Japanese version of the Kessler Psychological Distress Scale^40^ administered during the first trimester, available for 86 414 women.

### Main Analyses

The main analysis showed that the risks of preterm birth, moderate-to-late preterm birth, very preterm birth, and child born SGA increased with a higher level of maternal autistic traits (Table 2). In our crude model, a 1-SD increase in AQ-J10 score was associated with an increased risk of preterm birth (RR per 1-SD increase, 1.06; 95% CI, 1.03-1.09), moderate-to-late preterm birth (RR per 1-SD increase, 1.05; 95% CI, 1.01-1.08), very preterm birth (RR per 1-SD increase, 1.16; 95% CI, 1.07-1.26), and a child born SGA (RR per 1-SD increase, 1.04; 95% CI, 1.02-1.07). These risks showed little change after adjusting for covariates in model 2 and further adjusting for pregnancy complications in model 3; the highest RR was observed for very preterm birth (RR, 1.16; 95% CI, 1.06-1.26). To further illustrate the association between maternal autistic traits and outcomes, the estimated marginal probability of outcomes by the level of maternal autistic traits is shown in Figure. For all outcomes, a pattern of increasing probability was observed with a higher level of maternal autistic traits. Furthermore, postestimation analysis indicated that compared with women scoring below the AQ-J10 threshold, women scoring within the clinical range had a higher risk of preterm birth (RR, 1.16; 95% CI, 1.07-1.26), moderate-to-late preterm birth (RR, 1.12; 95% CI, 1.03-1.22), very preterm birth (RR, 1.49; 95% CI, 1.18-1.89), and a child born SGA (RR, 1.11; 95% CI, 1.04-1.19) (Table 3).

**Table 2.** Association Between Maternal Autistic Traits and Risk of Adverse Birth Outcomes

Outcome (gestational age)	Cases, No. (%) (N = 87 687)	RR (95% CI), per 1-SD increase in AQ-J10 score			
		Model 1	Model 2^a^	Model 3^b^
Preterm birth (<37 wk)	3941 (4.5)	1.06 (1.03-1.09)	1.06 (1.03-1.09)	1.06 (1.03-1.09)
Moderate-to-late preterm birth (32-36 wk)	3511 (4.0)	1.05 (1.01-1.08)	1.05 (1.01-1.08)	1.05 (1.01-1.08)
Very preterm birth (<32 wk)	430 (0.5)	1.16 (1.07-1.26)	1.16 (1.07-1.27)	1.16 (1.06-1.26)
Child born small for gestational age^c^	6722 (7.7)	1.04 (1.02-1.07)	1.04 (1.01-1.06)	1.04 (1.01-1.06)

Abbreviations: AQ-J10, short form of the Autism-Spectrum Quotient Japanese version; RR, relative risk.

^a^ Adjusted for maternal age at birth, maternal educational level, primiparous status, prepregnancy maternal body mass index, smoking during pregnancy, preexisting physical health condition, and child sex.

^b^ Adjusted for the covariates in model 2 and the presence or absence of gestational hypertension and gestational diabetes derived from medical records at birth.

^c^ Defined as birth weight below the tenth percentile of sex- and birth order–specific means for gestational age in Japan.

**Figure.**
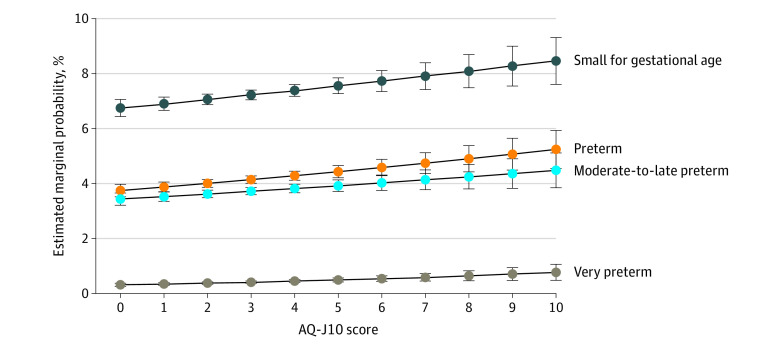
Estimated Marginal Probability of Adverse Birth Outcomes by Level of Maternal Autistic Traits The estimated marginal probability of adverse birth outcomes for each level of maternal autistic traits measured using the short form of the Autism-Spectrum Quotient Japanese version (AQ-J10) were derived from a model adjusting for maternal age at birth, maternal educational level, primiparous status, prepregnancy maternal body mass index, smoking during pregnancy, preexisting physical health condition, child sex, gestational hypertension, and gestational diabetes (model 3). Error bars indicate 95% CIs.

**Table 3.** Estimated Relative Risks of Adverse Birth Outcomes Comparing Mothers Scoring Above vs Below the Clinical Threshold for Autistic Traits

Outcome (gestational age)	Cases, No. (%)^a^		RR (95% CI)^b^
	Below AQ-J10 threshold (n = 85 337)	Within AQ-J10 clinical range (n = 2350)	
Preterm birth (<37 wks)	3824 (4.5)	117 (5.0)	1.16 (1.07-1.26)
Moderate to late preterm (32-36 wks)	3410 (4.0)	101 (4.3)	1.12 (1.03-1.22)
Very preterm (<32 wks)	414 (0.5)	16 (0.7)	1.49 (1.18-1.89)
Child born small for gestational age^c^	6527 (7.7)	195 (8.3)	1.11 (1.04-1.19)

Abbreviations: AQ-J10, short form of the Autism-Spectrum Quotient Japanese version; RR, relative risk.

^a^ The AQ-J10 score ranges from 0 to 10, and the clinical range was a score at or above the clinical cutoff of 7.

^b^ Relative risks and 95% CIs were obtained based on estimations from model 3 using the postestimation Stata command margins. The model was adjusted for maternal age at birth, maternal educational level, primiparous status, prepregnancy maternal body mass index, smoking during pregnancy, preexisting physical health condition, child sex, gestational hypertension, and gestational diabetes.

^c^ Defined as birth weight below the tenth percentile of sex and birth-order specific means for gestational age in Japan.

### Sensitivity Analysis

In our sensitivity analysis further adjusting for antenatal psychological distress preceding the measurement of maternal autistic traits, results were not altered (eTable 3 in Supplement 1). Analysis excluding women with a history of psychiatric conditions and those who took psychotropic medication during pregnancy yielded similar results (eTable 3 in Supplement 1).

## Discussion

In this nationwide prospective birth cohort study, we found that a higher level of maternal autistic traits in the general population was associated with an increased risk of preterm, moderate-to-late preterm, and very preterm births and a child born SGA, Risk was most pronounced for very preterm birth, for which a 1-SD increase in maternal autistic traits was associated with a 1.16-fold increased risk after adjusting for covariates and pregnancy complications. The risks of all birth outcomes were higher with a higher level of maternal autistic traits; for example, compared with women below the clinical range, women within the clinical range had greater risk of preterm births, moderate-to-late preterm births, very preterm births, and a child born SGA.

### Comparison With Previous Studies

To our knowledge, this is the first study to examine the association between maternal autistic traits and adverse birth outcomes in the general population. A previous study on pregnancy outcomes of women diagnosed with ASD found a 1.3-fold greater risk of preterm birth compared with those without a diagnosis using a Swedish medical record.^7^ The study also found a 1.23-fold greater risk of a child born SGA in autistic women not receiving medication.^7^ These results generally coincide with the results of our study. However, our study also showed that the risk of preterm births and a child born SGA was not limited to those diagnosed with ASD; the risks increased with a higher level of maternal autistic traits in the general population, with women scoring within the clinical range having the highest risks. Notably, the present study found that the increased risk of preterm birth was largely due to the increased risk of very preterm birth, which was not observed in the previous study.^7^ One explanation for this difference could be that women in the previous study had a formal ASD diagnosis, whereas only 18 women (0.02%) in the present study had an ASD diagnosis. Receiving a formal diagnosis has been reported to be associated with increased access to appropriate support and health care,^43,44^ which could have protected women against very preterm births in the previous study.^7^ The present study’s results show that women with a high level of autistic traits, particularly those within the clinical range and without a formal diagnosis, may have a high risk of adverse birth outcomes and require more attention and support during pregnancy.

Several explanations may account for the observed findings. First, previous studies have shown that women with developmental and intellectual disabilities, including ASD, were more likely to experience late entry to prenatal care and receive an inadequate number of hospital visits, which may be associated with increased risk of adverse birth outcomes.^2^ Studies on perinatal experiences of autistic mothers reported multiple barriers to receiving adequate perinatal care, such as communication barriers with health care practitioners, lack of tailored resources, and stigmatization.^45,46^ Women with a high level of autistic traits in the general population may experience health care disparities similar to those experienced by women diagnosed with ASD. Second, women with a higher level of autistic traits may experience more antenatal psychological distress, which if left untreated, is a potential risk factor for adverse birth outcomes.^47,48^ Compared with women with a lower level of autistic traits, women with a higher level of autistic traits had higher prevalence of factors associated with psychological distress, such as increased stressful life events,^49^ socioeconomic disadvantage,^50^ less help-seeking for mental health difficulties,^51^ and lack of social support during pregnancy.^36^ Therefore, women with a higher level of autistic traits may be more likely to experience psychological distress during pregnancy but may not ask for help, leading to an increased risk of adverse birth outcomes. Finally, a previous study reported that women with a higher level of autistic traits had inadequate dietary patterns, such as consuming fewer vegetables, fruits, or fish and less intake of certain nutrients during pregnancy,^52^ which may lead to fetal growth restriction and be associated with increased risk of preterm birth.^53^ Testing these hypotheses was beyond the scope of this study; however, examining these hypotheses in the future will offer additional evidence for preventive intervention.

### Strengths and Limitations

This study had many strengths, including the large sample size drawn from a nationwide cohort, the prospective longitudinal design of the study, and the use of medical records to derive data for birth outcomes, thus reducing the possibility of recall bias. However, this study had several limitations. First, the observational study design prevented the establishment of causal relationships. Second, although the proportion of women excluded due to missing responses was relatively small (5.0%) for a prospective cohort, this could have biased the observed association. Our sample bias analysis revealed that women scoring in the clinical range of the AQ-J10 and those with preterm births were more likely to be excluded from this study due to missing data, which could have underestimated the observed association. Third, although we measured maternal autistic traits using a validated questionnaire in the Japanese general population,^24^ reliance on self-report may be prone to response bias such as social desirability bias.^54^ We could not replicate the results by limiting the sample to women diagnosed with ASD because only 18 women reported being diagnosed with ASD in the present study. We acknowledge that autistic traits in the general population sometimes reflect social difficulties unrelated to ASD,^55^ such as those related to psychiatric conditions. However, the association remained after adjusting for antenatal psychological distress preceding the measurement of autistic traits or excluding women with a history of psychiatric conditions and those who reported taking psychotropic medication during pregnancy in our sensitivity analysis. In addition, the level of autistic traits in the general population is reported to be stable across time and is thus unlikely to be affected by pregnancy.^56^ Finally, there may have been unmeasured confounders, including genetic factors, underlying the observed association between maternal autistic traits and adverse birth outcomes. However, a previous study showed that maternal polygenetic risk scores for ASD were not associated with adverse birth outcomes, including preterm birth.^57^

## Conclusions

In this cohort study, a higher level of maternal autistic traits in the general population was associated with an increased risk of adverse birth outcomes, particularly very preterm birth. Health care practitioners should acknowledge the significant perinatal health disparity experienced by women with a high level of autistic traits, particularly those with autistic traits in the clinical range. Comprehensive antenatal care including the assessment of antenatal psychological distress and offering timely and tailored support that meets the needs of women should be provided inclusively regardless of whether a formal diagnosis of ASD is received. If provided, these approaches may improve maternal well-being with the potential to reduce the risk of adverse birth outcomes and have a downstream effect on child health and development.

## Notes

Supplement 1. **eMethods.** Assessment of covariates **eTable 1.** Descriptive characteristics by level of maternal autistic traits **eTable 2.** Comparison of characteristics of women included the analysis with those excluded **eTable 3.** Association between maternal autistic traits and adverse birth outcomes, further adjusted for antenatal psychological distress (Model S1) and excluding women with a history of psychiatric conditions or who took anti-psychiatric medication during pregnancy (Model S2) **eFigure 1.** Sample flowchart **eFigure 2.** Distribution of maternal autistic traits in the study sample

Supplement 2.
The Japan Environment and Children’s Study Group

Supplement 3.
Data Sharing Statement
