# Supplementary material for: Maternal Autistic Traits and Adverse Birth Outcomes
Source: JAMA Netw Open. 2024 Jan 23;7(1):e2352809. doi: 10.1001/jamanetworkopen.2023.52809 (PMC10807295; doi:10.1001/jamanetworkopen.2023.52809)
Supplement: Supplement 1. — eMethods. Assessment of covariates eTable 1. Descriptive characteristics by level of maternal autistic traits eTable 2. Comparison of characteristics of women included the analysis with those excluded eTable 3. Association between maternal autistic traits and adverse birth outcomes, further adjusted for antenatal psychological distress (Model S1) and excluding women with a history of psychiatric conditions or who took anti-psychiatric medication during pregnancy (Model S2) eFigure 1. Sample flowchart eFigure 2. Distribution of maternal autistic traits in the study sample [file jamanetwopen-e2352809-s001.pdf]

Supplemental Online Content

Hosozawa M, Cable N, Ikehara S, et al; Japan Environment and Children's Study Group. Maternal autistic traits and adverse birth outcomes. *JAMA Netw Open.* 2024;7(1):e2352809. doi:10.1001/jamanetworkopen.2023.52809

eMethods. Assessment of covariates

eTable 1. Descriptive characteristics by level of maternal autistic traits

eTable 2. Comparison of characteristics of women included the analysis with those excluded

eTable 3. Association between maternal autistic traits and adverse birth outcomes, further adjusted for antenatal psychological distress (Model S1) and excluding women with a history of psychiatric conditions or who took psychotropic medication during pregnancy (Model S2)

eFigure 1. Sample flow chart

eFigure 2. Distribution of maternal autistic traits in the study sample

This supplemental material has been provided by the authors to give readers additional information about their work.

© 2024 Hosozawa M et al. *JAMA Network Open.*

eMethod 1. Assessment of covariates

1) Assessment of antenatal psychological distress

Antenatal psychological distress was assessed during the first trimester by using the Japanese version of the Kessler Psychological Distress Scale (K6+).[1] The K6+ is a validated six-item self-rated psychological distress questionnaire that measures symptoms of depression and anxiety over the last 30 days (range 0-24, higher scores indicate more severe distress). [2] The scale is reported to predict mood or anxiety disorder according to the Diagnostic and Statistical Manual of Mental Disorders, Fourth Edition (DSM-IV).[1]

1. Furukawa TA, Kawakami N, Saitoh M, et al. The performance of the Japanese version of the K6 and K10 in the World Mental Health Survey Japan. *Int J Methods Psychiatr Res*. 2008;17(3):152-8. doi:10.1002/mpr.257
2. Kessler RC, Green JG, Gruber MJ, et al. Screening for serious mental illness in the general population with the K6 screening scale: results from the WHO World Mental Health (WMH) survey initiative. *Int J Methods Psychiatr Res*. Jun 2010;19 Suppl 1:4-22. doi:10.1002/mpr.310

2) Assessment of pre-existing health conditions, pre-existing psychiatric conditions and psychotropic medication

Information on pre-existing physical and psychiatric conditions was obtained from maternal self-reports during pregnancy. The use of psychotropic medication during pregnancy was obtained by interviewing the mothers by Research Coordinators.

Condition	Included diagnosis or medication
Pre-existing physical health condition	Hypertension<br>Diabetes mellitus<br>Asthma<br>Endocrine condition: Hyperthyroidism, Hypothyroidism<br>Reproductive health condition: Endometriosis, Uterine fibroids, Adenomyosis uteri
Pre-existing psychiatric condition	Depressive disorder<br>Anxiety disorder<br>Schizophrenia<br>Epilepsy<br>Attention Deficit and Hyperactivity Disorder<br>Learning disorder<br>Autism Spectrum Disorder<br>Other developmental disorders
Psychotropic medication	Selective Serotonin Reuptake Inhibitors (SSRI)<br>Antidepressants other than SSRI<br>Anxiolytics<br>Antipsychotics<br>Valproic acid<br>Anti-epileptic drugs other than valproic acid<br>Lithium carbonate

© 2024 Hosozawa M et al. *JAMA Network Open.*

eTable 1. Descriptive characteristics by level of maternal autistic traits

Characteristic	No. (%) of participants	
	Maternal autistic traits groups [a]	
	Below threshold (n = 85337, 97.3%)	Clinical-range (n = 2350, 2.7%)
Maternal age at delivery, mean (SD), years	31.2 (5.0)	30.6 (5.2)
Maternal autistic traits, mean (SD)[a]	2.7 (1.5)	7.4 (0.6)
Highest maternal education level		
High school graduate or below	30509 (35.8)	1020 (43.4)
Vocational school or junior college	36093 (42.3)	864 (36.8)
College graduate and above	18735 (22.0)	466 (19.8)
Primiparous	37206 (43.6)	1164 (49.5)
Pre-pregnancy body mass index, mean (SD)	21.2 (3.3)	21.3 (3.2)
Pre-existing physical health condition	17858 (20.9)	504 (21.4)
Ever diagnosed with ASD	7 (0.01)	11 (0.5)
Had smoked during pregnancy	4491 (5.3)	154 (6.6)
Gestational hypertension	2692 (3.2)	73 (3.1)
Gestational diabetes mellitus	2314 (2.7)	50 (2.1)
Child sex (Male)	43767 (51.3)	1210 (51.5)
Mean antenatal psychological distress[b]	3.6 (3.8)	5.5 (4.8)

ASD = autism spectrum disorder, SD = standard deviation.

[a] Measured using the short form of the Autism Spectrum Quotient Japanese version (AQ-J10) administered during the second and third trimesters. The Clinical-range represents women who scored above the clinical cutoff of seven.

[b] Measured using the Kessler Psychological Distress Scale (K6+) administered during the first trimester, available for 86414 women.

© 2024 Hosozawa M et al. *JAMA Network Open.*

eTable 2. Comparison of characteristics of women included the analysis with those excluded [a]

Characteristic	No. (%) of Participants (N = 92279)		P-value [b]
	Analytic sample (n = 87687)	Non-analytic sample (n = 4592)	
Maternal age at delivery, mean (SD), years	31.2 (5.0)	30.5 (5.6)	< 0.001
Maternal autistic traits, mean (SD) [c]	2.8 (1.7)	2.8 (1.8)	0.44
Autistic traits in the clinical-range (≥7) [c]	2,350 (2.7)	26 (4.0)	0.037
Highest maternal education level			
High school graduate or below	31529 (36.0)	1099 (44.0)	< 0.001
Vocational school or junior college	36957 (42.1)	995 (39.8)	
College graduate and above	19201 (21.9)	403 (16.1)	
Primiparous			
No	49317 (56.2)	2706 (60.6)	< 0.001
Yes	38370 (43.8)	1756 (39.4)	
Pre-pregnancy body mass index, mean (SD)	21.2 (3.3)	21.4 (3.6)	< 0.001
Pre-existing physical health condition			
No	69325 (79.1)	3722 (81.1)	0.001
Yes	18362 (20.9)	870 (18.9)	
Ever diagnosed with ASD			
No	87276 (100.0)	3794 (100.0)	0.38
Yes	18 (0.02)	0 (0)	
Had smoked during pregnancy			
No	83042 (94.7)	3366 (91.2)	< 0.001
Yes	4645 (5.3)	323 (8.8)	
Gestational hypertension			
No	84922 (96.8)	4429 (96.5)	0.14
Yes	2765 (3.2)	163 (3.5)	
Gestational diabetes mellitus			
No	85323 (97.3)	4468 (97.3)	0.99
Yes	2364 (2.7)	124 (2.7)	
Child sex			
Female	42710 (48.7)	2219 (48.4)	0.71
Male	44977 (51.3)	2363 (51.6)	
Antenatal psychological distress, mean (SD)	3.7 (3.8)	3.9 (4.2)	< 0.001
Gestational age groups			
Full-term	83746 (95.5)	4221 (91.9)	< 0.001
Moderate-to-late preterm	3511 (4.0)	255 (5.6)	
Very preterm	430 (0.5)	116 (2.5)	
Small-for-gestational age			
No	80965 (92.3)	4158 (91.9)	0.30
Yes	6722 (7.7)	366 (8.1)	

ASD = autism spectrum disorder, SD = standard deviation.
[a] N varies due to missing values.
[b] P-values for group differences were obtained using t-tests for continuous variables and chi-squared tests for categorical variables.
[c] Measured using the short form of Autism Spectrum Quotient Japanese version (AQ-J10) administered during the second and third trimesters. The Clinical-range represents women who scored above the clinical cutoff of seven.

© 2024 Hosozawa M et al. *JAMA Network Open.*

eTable 3. Association between maternal autistic traits and adverse birth outcomes, further adjusted for antenatal psychological distress (Model S1) and excluding women with a history of psychiatric conditions or who took psychotropic medication during pregnancy (Model S2)

Outcomes	Case, No. (%)	RR (95% CI) [a]	Case, No. (%)	RR (95% CI) [a]
	**Model S1: Further adjusted for antenatal psychological distress (n = 86414) [b,c]**		**Model S2: Excluding women with a history of psychiatric conditions or who took psychotropic medication during pregnancy (n = 82553) [d,e]**	
Preterm (< 37 weeks)	3877 (4.5)	1.05 (1.02-1.08)	3646 (4.4)	1.06 (1.03-1.09)
Moderate-to-late preterm (32-36 weeks)	3453 (4.0)	1.04 (1.00-1.07)	3246 (3.9)	1.04 (1.01-1.08)
Very preterm (<32 weeks)	424 (0.5)	1.16 (1.06-1.27)	400 (0.5)	1.18 (1.08-1.29)
Small-for-gestational age	6624 (7.7)	1.04 (1.01-1.06)	6315 (7.7)	1.04 (1.01-1.06)

RR= relative risk. CI= confidence interval.

[a] The results are presented for a 1 SD increase in maternal autistic traits.

[b] Adjusted for maternal age at birth, maternal education level, primiparous status, pre-pregnancy maternal body mass index, smoking during pregnancy, pre-existing physical health condition, child sex, gestational hypertension, gestational diabetes mellitus, and antenatal psychological distress.

[c] Antenatal psychological distress was measured using the Kessler Psychological Distress Scale (K6+) during the first trimester.

[d] History of psychiatric conditions was measured based on self-report, and the use of psychotropic medication during pregnancy was obtained from maternal interviews by research coordinators (See eMethod2 for details).

[e] Adjusted for maternal age at birth, maternal education level, primiparous status, pre-pregnancy maternal body mass index, smoking during pregnancy, pre-existing physical health condition, child sex, gestational hypertension, and gestational diabetes mellitus.

© 2024 Hosozawa M et al. *JAMA Network Open.*

eFigure 1. Sample flow chart

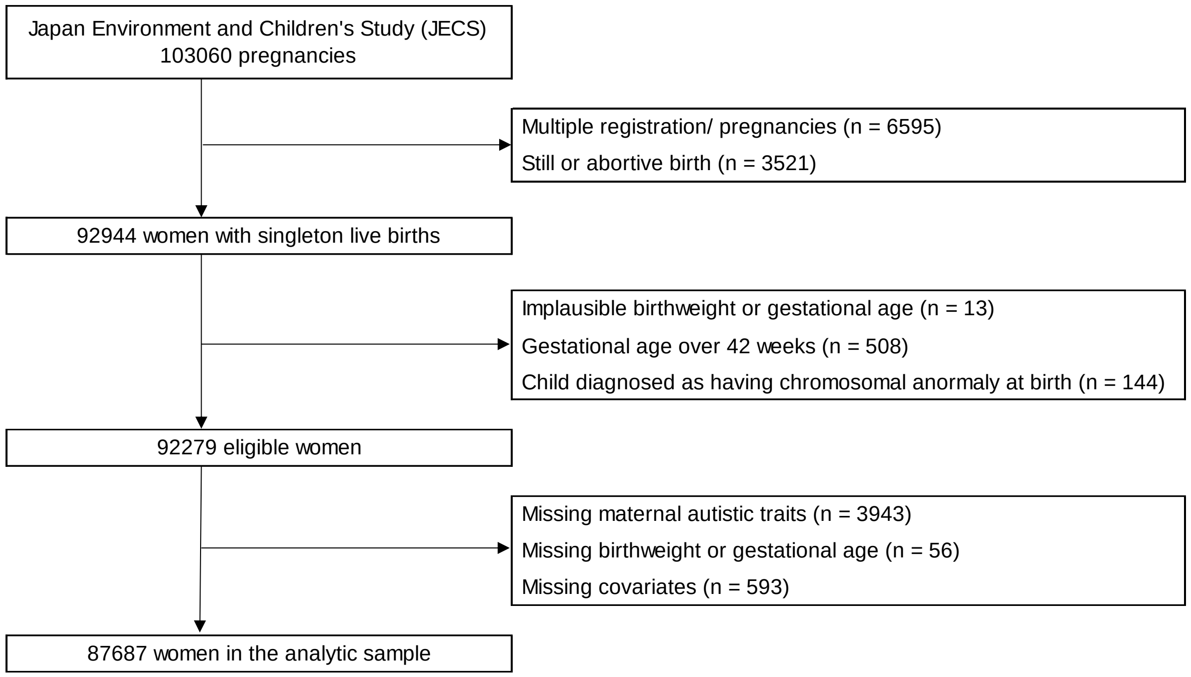

© 2024 Hosozawa M et al. *JAMA Network Open.*

eFigure 2. Distribution of maternal autistic traits in the study sample (N = 87687)

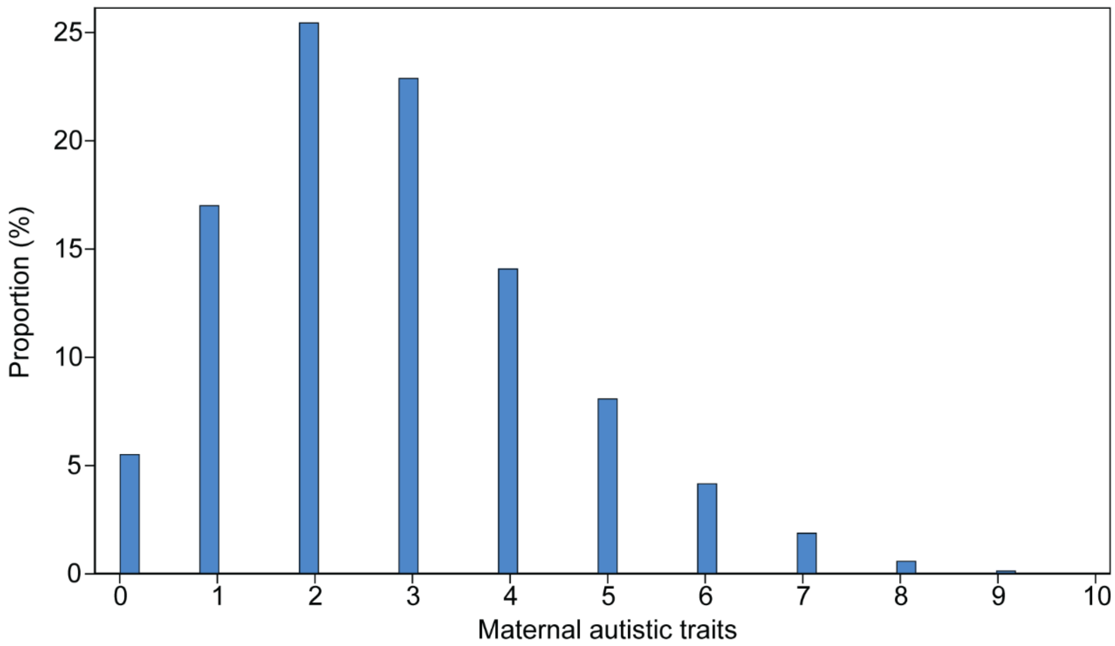

The proportion of women scoring each score category for the short form of the Autism Spectrum Quotient Japanese version (AQ-J10, range 0-10) administered during the second and third trimesters is shown.

© 2024 Hosozawa M et al. *JAMA Network Open.*
