# Supplementary material for: Maternal Autistic Traits and Adverse Birth Outcomes
Source: JAMA Netw Open. 2024 Jan 23;7(1):e2352809. doi: 10.1001/jamanetworkopen.2023.52809 (PMC10807295; doi:10.1001/jamanetworkopen.2023.52809)
Supplement: Supplement 2. — The Japan Environment and Children’s Study Group [file jamanetwopen-e2352809-s002.pdf]

Supplemental Online Content: Nonauthor Collaborators
*First name, last name, and suffix (if applicable) are required and will appear in PubMed.

| *Group Name(s): The Japan Environment and Children's Study Group | | | | | | | |
|---|---|---|---|---|---|---|---|
| ***First Name and Middle Initial(s)** | ***Last Name** | ***Suffix (eg, Jr, III)** | Academic Degrees | Institution | Location (city, state/province, country) | Role or Contribution, eg, chair, principal investigator | Group (if more than 1 Group listed in the byline) and/or Subgroup (eg, Steering Committee) |
| Michihiro | Kamijima | | MD PhD | Nagoya City University | Nagoya, Aichi, Japan | Principal investigator | |
| Shin | Yamazaki | | PhD | National Institute for Environmental Studies | Tsukuba, Ibaraki, Japan | Project design, data collection and review | |
| Yukihiro | Ohya | | MD PhD | National Center for Child Health and Development | Tokyo, Japan | Project design, data collection and review | |
| Reiko | Kishi | | MD PhD | Hokkaido University | Sapporo, Hokkaido, Japan | Project design, data collection and review | |
| Nobuo | Yaegashi | | MD PhD | Tohoku University | Sendai, Miyagi, Japan | Project design, data collection and review | |
| Koichi | Hashimoto | | MD PhD | Fukushima Medical University | Fukushima, Japan | Project design, data collection and review | |
| Chisato | Mori | | MD PhD | Chiba University | Chiba, Japan | Project design, data collection and review | |
| Shuichi | Ito | | MD PhD | Yokohama City University | Yokohama, Kanagawa, Japan | Project design, data collection and review | |
| Zentaro | Yamagata | | MD PhD | University of Yamanashi | Chuo, Yamanashi, Japan | Project design, data collection and review | |
| Hidekuni | Inadera | | MD PhD | University of Toyama | Toyama, Japan | Project design, data collection and review | |
| Takeo | Nakayama | | MD PhD | Kyoto University | Kyoto, Japan | Project design, data collection and review | |
| Tomotaka | Sobue | | MD PhD | Osaka University | Suita, Osaka, Japan | Project design, data collection and review | |
| Masayuki | Shima | | MD PhD | Hyogo Medical University | Nishinomiya, Hyogo, Japan | Project design, data collection and review | |
| Seiji | Kageyama | | MD PhD | Tottori University | Yonago, Tottori, Japan | Project design, data collection and review | |
| Narufumi | Suganuma | | MD PhD | Kochi University | Nankoku, Kochi, Japan | Project design, data collection and review | |

Supplemental Online Content: Nonauthor Collaborators
*First name, last name, and suffix (if applicable) are required and will appear in PubMed.

| ***First Name and Middle Initial(s)** | ***Last Name** | ***Suffix (eg, Jr, III)** | Academic Degrees | Institution | Location (city, state/province, country) | Role or Contribution, eg, chair, principal investigator | Group (if more than 1 Group listed in the byline) and/or Subgroup (eg, Steering Committee) |
|---|---|---|---|---|---|---|---|
| Shoichi | Ohga | | MD PhD | Kyushu University | Fukuoka, Japan | Project design, data collection and review | |
| Takahiko | Katoh | | MD PhD | Kumamoto University | Kumamoto, Japan | Project design, data collection and review | |
