# Supplementary material for: Maternal Autistic Traits and Adverse Birth Outcomes
Source: JAMA Netw Open. 2024 Jan 23;7(1):e2352809. doi: 10.1001/jamanetworkopen.2023.52809 (PMC10807295; doi:10.1001/jamanetworkopen.2023.52809)
Supplement: Supplement 3. — Data Sharing Statement [file jamanetwopen-e2352809-s003.pdf]

Data Sharing Statement

Hosozawa. Maternal Autistic Traits and Adverse Birth Outcomes. *JAMA Netw Open*. Published January 23, 2024. doi:10.1001/jamanetworkopen.2023.52809

Data
Data available: No

Additional Information
Explanation for why data not available: Data are unsuitable for public deposition due to ethical restrictions and legal framework of Japan. It is prohibited by the Act on the Protection of Personal Information (Act No. 57 of 30 May 2003, amendment on 9 September 2015) to publicly deposit the data containing personal information. Ethical Guidelines for Medical and Health Research Involving Human Subjects enforced by the Japan Ministry of Education, Culture, Sports, Science and Technology and the Ministry of Health, Labour and Welfare also restricts the open sharing of the epidemiologic data. All inquiries about access to data should be sent to: jecs-en@nies.go.jp. The person responsible for handling enquiries sent to this e-mail address is Dr Shoji F. Nakayama, JECS Programme Office, National Institute for Environmental Studies.
